# The impacts of health insurance on financial strain for people with chronic diseases

**DOI:** 10.1186/s12889-021-11075-2

**Published:** 2021-05-29

**Authors:** Zixuan Peng, Li Zhu

**Affiliations:** 1 Institute of Health Policy, Management & Evaluation, Dalla Lana School of Public Health, University of Toronto, Toronto, Canada; 2 School of Political Science and Public Administration, Guangxi University for Nationalities, Nanning, China

**Keywords:** Chronic diseases, Health insurance, Financial strain

## Abstract

**Background:**

Due to ongoing expenses for both short-term and long-term needs for health services, people with chronic diseases tend to struggle with financial hardship. Health insurance is employed as a useful tool in aiding people to solve such financial strain. This study aims to examine and compare the impacts of public and private health insurance on solving financial barriers for people with chronic diseases.

**Methods:**

This research obtained an outpatient sample consisted of 1739 individuals and an inpatient sample consisted of 1034 individuals. We employed a *Chi-square* test and a two-sample *T-*test to explore differences in financial strain and insurance status between people with chronic diseases and those without. Then we adopted binary logistic regression technique to assess the impacts of different types of health insurance on outpatient and inpatient financial strain for people with chronic diseases.

**Results:**

Our research has five key findings: first, people with chronic diseases were more likely to experience both the outpatient and inpatient financial strain (*P* < 0.01); second, public health insurance was found to reduce the outpatient financial strain; third, private health insurance was found to positively associate with inpatient financial barriers; fourth, Urban Employment Insurance (UEI) was expected to reduce both the outpatient and inpatient financial barriers, while self-paid private insurance (SPI) was positively associated with inpatient financial barriers; and fifth, income was identified as a positive predictor of having outpatient and inpatient financial strain.

**Conclusions:**

Public health insurance has the potential to reduce the outpatient financial strain for people with chronic diseases. Private health insurance was identified as a positive predictor of inpatient financial strain for people with chronic diseases. Policy should be proposed to promote the capacity of public health insurance and explore the potential effects of private health insurance on solving the inpatient financial barriers faced by people with chronic diseases in China.

## Background

Non-communicable diseases (NCDs), also known as chronic diseases, tend to be of long duration and are the result of a combination of genetic, physiological, environmental and behaviors factors [1]. NCDs are the leading cause of death globally and nearly 80% of NCDs occur in low- and middle-income countries [2]. In China, the prevalence of NCDs in 2003 was 12.33%, while this figure had doubled to 24.52% in 2013 [3]. People with NCDs tended to have financial burden due to ongoing expenses for both short- and long-term needs for health services [4–8]. Such financial strain may force individuals to choose among competing demands, such as skipping meals to pay for medicines or delaying health care visits to pay for utilities [9], which in turn may result in hospitalization and increased mortality [10, 11].

Health insurance is employed as a useful tool to protect individuals against the risks of large unexpected medical expenditures [12, 13]. Previous studies have already demonstrated that public health insurance had the potential to offer substantial protection from medical financial risks [12, 14]. However, public health insurance is always partial, since it concerns either a limited basket of care (e.g. in Canada where drugs are out of the public system or in Spain and in the UK where services provided by private physicians are uncovered), a limited population (as in the US where public coverage only covers old, vulnerable and poor populations) or since it lets copayments on a quite large basket of care through coinsurance rates and deductibles (as in Belgium, in France or in Switzerland) [15, 16]. Private health insurance, in this circumstance, often functions to supplement public health insurance systems to provide financial protection [17, 18].

The positive impacts of health insurance on alleviating the financial strain for people with chronic diseases have been extensively reported [19–21]. But prior studies have also presented opposite findings. For instance, Cagle et al. found that despite being well insured, a quarter of respondents reported that the cost of care was still a major financial burden [22]. Likewise, Sun et al. conducted a study in rural China, concluding that the insurance scheme offered only a very limited degree of financial protection for people with chronic diseases [21]. Meanwhile, it was found that there exist variations in the impacts of various types of public health insurance on reducing the financial strain for people with chronic diseases [23]. Moreover, although private health insurance reforms with the goal of helping people with chronic diseases to better manage their medical expenditure, the longer term benefits of these private insurance schemes remain to be properly assessed [24]. Hence, there are opportunities to contribute to the literature through examining the impacts of different types of health insurance on solving the financial strain for people with chronic diseases in China.

The purpose of this research is twofold: first, to explore whether health insurance could reduce the financial barriers for people with chronic diseases in China; and second, to compare the impacts of different health insurance types on financial barriers. This study is structured as follows: In Section 2, we introduce data sources, study variables and the statistical methods employed. Our research results are outlined in Section 3. We move on with discussion in Section 4 before making conclusions and potential policy suggestions in Section 5.

## Methods

### Data source

Data of this research was extracted from China Health and Retirement Longitudinal Study (CHARLS) which contains high-quality micro-level data of individuals aged ≥45 years of age in China. The CHARLS aims to collect a high quality nationally representative sample of Chinese residents ages 45 and older to serve the needs of scientific research on the elderly. The CHARLS includes about 10,000 households and 17,500 individuals in 150 counties/districts and 450 villages/resident committees. Details of the design of CHARLS are available on the website: http://charls.pku.edu.cn/index/en.html.

In our research, participants were identified as having chronic diseases if they reported having at least one of the chronic conditions below: hypertension, dyslipidemia, diabetes or high blood sugar, cancer or malignant tumor, chronic lung diseases, liver disease, heart attack, stroke, kidney disease, stomach or other digestive diseases, and emotional, nervous, or psychiatric problems. After dropping sample elements that reported “don’t know about the answer”, “refused to answer the questions”, or had “other special missing responses”, we finally obtained a sample consisted of 1739 individuals who had outpatient visits and a sample consisted of 1034 individuals who had inpatient visits in 2015.

### Study variables

Financial strain was the outcome of our interest. Financial strain was measured by a binary variable through answering the following question: did you not seek outpatient or inpatient health services mainly due to not having enough money [25]? Our research included various types of health insurance as the independent variables for further investigation. To realize the goals that everyone will have access to health care and poor people can afford the medical costs, the Chinese government has established a multi-level medical security system, including the universal health care system, the commercial medical insurance, and a medical charity aid scheme [26]. The former two constitute the health insurance system in China, including the public health insurance launched by the government and private health insurance paid by employers or people themselves; medical charity is a form of public health assistance, which is not the focus of our research and therefore was excluded. Specifically, public health insurance in our research included Urban Employee Basic Medical Insurance that covers urban employees (UEI, launched in 1998) [27], New Rural Cooperative Medical Insurance that covers rural residents (NRCI, launched in 2003) [28], and Urban Resident Basic Medical Insurance that covers urban residents (URI, launched in 2007) [29]. Meanwhile, private health insurance was categorized into employer-paid private insurance (EPI) and self-paid private insurance (SPI) [30]. According to the China Social Security Law, UEI is a mandatory health insurance scheme funded by employees, employers and governments, whereas URI and NRCI are voluntary schemes [31]. Overall, although the three public health insurance schemes cover the same service packages prescribed by the government, their premiums and the reimbursement ratios are different. More details about these different insurance schemes were reported in Table 1.

**Table 1:** Health insurance schemes in China in 2015

	***UEI*** ^***b***^	***URI***	***NRCI***	***EMI***	***SPI***
Total Premium per Person	CNY 3143	CNY 530.7	CNY 490.3	–	–
Contribution of government	–	CNY 380 per capita per year	CNY 380 per capita per year	–	–
Contribution of employers	About 6–8% of average wage	–	–	Depends	–
Contribution of individuals	About 2% of salary	CNY 120 per capita per year	CNY 90 per capita per year	Depends	Depends
Service Coverage	Social account ^c^ for inpatient care and individual account for outpatient care	Social account for inpatient care and household account for outpatient care	Social account for inpatient care and household account for outpatient care	Depends	Depends

Source: ^a^ Data was extracted from the National Health Statistics Annual Report, National Basic Medical Security Statistics Annual Report, and China Statistical Yearbook 2016; ^b^ UEI, URI, NRCI, EMI, and SPI represents Urban Employee Basic Medical Insurance, Urban Resident Basic Medical Insurance, New Rural Cooperative Medical Insurance, employer-paid private insurance, and self-paid private insurance, respectively; ^c^ Individual accounts are funded by employee and employer contributions, while social accounts are funded by employer contributions

Following Andersen healthcare utilization model [32], additional covariates considered by our research included: 1) Predisposing factors, including age, gender (female or male) and marital status (married with spouse present, married but not living with spouse, separated, divorced, widowed, never married, or cohabited) [19]; 2) Enabling factors, including education (less than lower secondary, upper secondary and vocational training, or tertiary education), “hukou” type (agricultural “hukou”, non-agricultural “hukou”, unified residence “hukou”, or do not have “hukou”), income, and household size [33]; and 3) Need factors, including self-reported health status (excellent, very good, good, fair, or poor) [19], reporting having at least one functional limitation (physical disabilities, brain damage/mental retardation, vision problem, hearing problem and speech impediment), number of chronic diseases (hypertension, dyslipidemia, diabetes or high blood sugar, cancer or malignant tumor, chronic lung diseases, liver disease, heart attack, stroke, kidney disease, stomach or other digestive disease, and emotional, nervous, or psychiatric problems) [33].

### Statistical analyses

Our research undertook a series of statistical analyses: First, we examined the distribution of variables in group with chronic diseases and group in the absence of chronic diseases. Categorical variables were reported as counts and percentages, while continuous variables were reported as mean and standard deviation. The distribution of variables in the two groups was assessed using a χ2 test (for categorical variables) and a two-sample *t* test (for continuous variables). Second, since our dependent variables were binary, we constructed binary logistic regression models to investigate the impacts of different types of health insurance on financial strain. Specifically, our research first constructed a public health insurance model and a private health insurance model to compare their impacts on financial strain. Meanwhile, since diverse types of health insurance differ in their premiums, service packages, and reimbursement ratios, we further constructed two models, with each including separate types of public or private health insurance as our independent variables. Third, considering the possible endogenous relationship between having insurance and financial strain, we used Propensity score matching (K-nearest neighbor matching method, caliper value of 20%) to obtain matched samples [34]. Our research conducted a binary regression where whether having insurance was included as the dependent variable and all the covariates identified by our study were included as the independent variables. Specifically, voluntary health insurance schemes (including URI, NRCI, EPI, and SPI) were included as our dependent variables, while mandatory health insurance scheme (UEI) was excluded by our study. Then the treatment effects of having insurance on financial strain were assessed using two samples with similar characteristics in covariates but having different insurance status. McFadden’s *R*^*2*^ and the value of Area Under the Curve (AUC) were calculated to measure the goodness of fit and predictive power of the logistic regression models, respectively. The AUC value of 0.50 to 0.65 represents little predictive power, 0.65 to 0.80 means moderate predictive power, and 0.80 to 1.00 denotes strong predictive power [35]. All the analyses of our research were conducted using *R* statistical software [36].

## Results

### Descriptive analysis

Table 2 reports the differences between groups with chronic diseases and those without chronic diseases. Among 1739 people with chronic diseases, 16.16% (*N* = 281) reported having outpatient financial strain. People with chronic diseases (*N* = 234; 18.00%) were more likely to have outpatient financial strain than people without chronic diseases (*N* = 47; 10.70%) (*P* <  0.01). Meanwhile, people with chronic diseases were more likely to have URI while less likely to have NRCI than people without chronic diseases (*P* <  0.05). Of 1034 people with chronic diseases, 54.26% (*N* = 561) reported having inpatient financial strain. People with chronic diseases (*N* = 473; 56.90%) were more likely to have inpatient financial strain than those in the absence of chronic diseases (*N* = 88; 43.30%) (*P* <  0.01). In the meantime, people with chronic diseases were less likely to have NRCI than people without chronic diseases (*P* = 0.01).

**Table 2:** Differences in groups by chronic diseases

***Variables***	***Chronic diseases (outpatient sample)***				***Chronic diseases (inpatient sample)***			
Number	Categories	No = 441	Yes = 1298	*P-*value	Categories	No = 203	Yes = 831	*P-*value
Financial strain (%)	No = 1458	394 (89.3)	1064 (82.0)	< 0.001	No = 473	115 (56.7)	358 (43.1)	0.001
	Yes = 281	47 (10.7)	234 (18.0)		Yes = 561	88 (43.3)	473 (56.9)	
Public insurance (%)	No = 180	38 (8.6)	142 (10.9)	0.196	No = 84	15 (7.4)	69 (8.3)	0.776
	Yes = 1559	403 (91.4)	1156 (89.1)		Yes = 950	188 (92.6)	762 (91.7)	
Private insurance (%)	No = 1707	430 (97.5)	1277 (98.4)	0.328	No = 1016	196 (96.6)	820 (98.7)	0.076
	Yes = 32	11 (2.5)	21 (1.6)		Yes = 18	7 (3.4)	11 (1.3)	
UEI (%)	No = 1564	387 (87.8)	1177 (90.7)	0.095	No = 954	189 (93.1)	765 (92.1)	0.724
	Yes = 175	54 (12.2)	121 (9.3)		Yes = 80	14 (6.9)	66 (7.9)	
URI (%)	No = 1653	429 (97.3)	1224 (94.3)	0.018	No = 988	197 (97.0)	791 (95.2)	0.337
	Yes = 86	12 (2.7)	74 (5.7)		Yes = 46	6 (3.0)	40 (4.8)	
NRCI (%)	No = 612	137 (31.1)	475 (36.6)	0.041	No = 318	47 (23.2)	271 (32.6)	0.011
	Yes = 1127	304 (68.9)	823 (63.4)		Yes = 716	156 (76.8)	560 (67.4)	
EPI (%)	No = 1732	439 (99.5)	1293 (99.6)	1.000	No = 1033	203 (100.0)	830 (99.9)	1.000
	Yes = 7	2 (0.5)	5 (0.4)		Yes = 1	0 (0.0)	1 (0.1)	
SPI (%)	No = 1720	437 (99.1)	1283 (98.8)	0.866	No = 1020	197 (97.0)	823 (99.0)	0.062
	Yes = 19	4 (0.9)	15 (1.2)		Yes = 14	6 (3.0)	8 (1.0)	

Table 3 reports the differences between group with financial strain and group without financial strain. Among people who reported having outpatient financial barriers, 84.30% (*N* = 237) had public health insurance, which was smaller than the figure for people without outpatient financial strain (*N* = 1322; 90.70%) (*P* <  0.01). Meanwhile, it was found that people with outpatient or inpatient financial barriers were less likely to have UEI (*P* <  0.01). Moreover, differences in marital status, income, health status, functional limitation, and number of chronic diseases were found in groups classified by whether having outpatient financial barriers (*P* <  0.05). Meanwhile, differences in age, education, “hukou” type, income, health status, and number of chronic diseases were found in groups classified by whether having inpatient financial barriers (*P* <  0.05).

**Table 3:** Descriptive analysis of the outpatient and inpatient sample

		***Outpatient financial strain***			***Inpatient financial strain***		
		No = 1458	Yes = 281	*P-*value	No = 473	Yes = 561	*P-*value
Private insurance (%)	Have private insurance	27 (1.9)	5 (1.8)	1.000	8 (1.7)	10 (1.8)	1.000
Public insurance(%)	Have public insurance	1322 (90.7)	237 (84.3)	0.002	439 (92.8)	511 (91.1)	0.370
UEI	Have UEI	168 (11.5)	7 (2.5)	< 0.001	54 (11.4)	26 (4.6)	< 0.001
URI	Have URI	70 (4.8)	16 (5.7)	0.630	22 (4.7)	24 (4.3)	0.890
NRCI	Have NRCI	947 (65.0)	180 (64.1)	0.826	322 (68.1)	394 (70.2)	0.496
EPI	Have EPI	5 (0.3)	2 (0.7)	0.704	0 (0.0)	1 (0.2)	1.000
SPI	Have SPI	18 (1.2)	1 (0.4)	0.325	7 (1.5)	7 (1.2)	0.959
Age		61.01 (10.62)	61.49 (9.94)	0.484	62.27 (10.17)	60.91 (8.84)	0.022
Gender	Female	821 (56.3)	165 (58.7)	0.496	264 (55.8)	339 (60.4)	0.151
	Male	637 (43.7)	116 (41.3)		209 (44.2)	222 (39.6)	
Marital status	Married	1118 (76.7)	204 (72.6)	0.001	373 (78.9)	442 (78.8)	0.252
	Partnered	113 (7.8)	13 (4.6)		25 (5.3)	24 (4.3)	
	Separated	2 (0.1)	1 (0.4)		1 (0.2)	1 (0.2)	
	Divorced	9 (0.6)	4 (1.4)		5 (1.1)	5 (0.9)	
	Widowed	205 (14.1)	50 (17.8)		68 (14.4)	79 (14.1)	
	Never married	11 (0.8)	9 (3.2)		1 (0.2)	10 (1.8)	
Household size		3.11 (1.42)	2.93 (1.33)	0.053	2.90 (1.29)	3.01 (1.30)	0.160
Education	Less than lower secondary	1306 (89.6)	263 (93.6)	0.082	406 (85.8)	527 (93.9)	< 0.001
	Upper secondary and vocational training	131 (9.0)	17 (6.0)		54 (11.4)	31 (5.5)	
	Tertiary	21 (1.4)	1 (0.4)		13 (2.7)	3 (0.5)	
“Hukou” type	Agricultural “hukou”	1188 (81.5)	245 (87.2)	0.119	378 (79.9)	487 (86.8)	0.005
	Non-agricultural “hukou”	245 (16.8)	32 (11.4)		87 (18.4)	71 (12.7)	
	Unified residence “hukou”	22 (1.5)	4 (1.4)		7 (1.5)	1 (0.2)	
	Do not have “hukou”	3 (0.2)	0 (0.0)		1 (0.2)	2 (0.4)	
Income		3717.21 (16,089.42)	1247.16 (6016.25)	0.011	2556.54 (8706.01)	905.78 (3661.76)	< 0.001
Health status	Excellent	5 (0.3)	0 (0.0)	< 0.001	1 (0.2)	2 (0.4)	< 0.001
	Very good	50 (3.4)	5 (1.8)		18 (3.8)	11 (2.0)	
	Good	108 (7.4)	13 (4.6)		18 (3.8)	12 (2.1)	
	Fair	852 (58.4)	118 (42.0)		230 (48.6)	206 (36.7)	
	Poor	443 (30.4)	145 (51.6)		206 (43.6)	330 (58.8)	
Functional limitation	Have functional limitation	280 (19.2)	78 (27.8)	0.002	102 (21.6)	148 (26.4)	0.084
Number of chronic diseases		2.08 (1.97)	2.87 (2.16)	< 0.001	2.46 (2.12)	2.96 (2.19)	< 0.001

Number and percentage were reported for categorical variables, while mean and standard deviation were reported for continuous variables

### The impacts of health insurance on financial strain

Table 4 reports the results of binary logistic regression where public and private health insurance were included as the independent variables. Public health insurance was found to negatively associate with outpatient financial strain. Specifically, the odds for people with public health insurance to have outpatient financial strain were substantively lower than people without public health insurance (OR: 0.58; 95% CI: 0.34–3.55). In contrast, private health insurance was found to positively associate with inpatient financial strain. Specifically, people with private health insurance had 6.25 times the odds (95% CI: 1.33–52.22) of having inpatient financial barriers, relative to those without private health insurance. Meanwhile, age, marital status, income, functional limitation, and number of chronic diseases were statistically significant predictors of having outpatient financial barriers, while education, “hukou” type, income, and functional limitation were statistically significant predictors of having inpatient financial barriers. The McFadden’s *R*^*2*^ of the outpatient and the inpatient regression model was 9.27 and 6.77%, respectively. Meanwhile, it was found that both the outpatient and the inpatient regression model have moderate predictive power (AUC = 0.65).

**Table 4:** The impacts of health insurance on financial strain for people with chronic diseases

	***Outpatient financial strain***				***Inpatient financial strain***			
	***Estimate***	***Std.Error***	***Z-value***	***OR***	***Estimate***	***Std.Error***	***Z-value***	***OR***
Intercept	−14.520	1281.000	−0.011	0.000	16.470	624.200	0.026	14,217,725.233
**Private health insurance**	0.202	0.587	0.344	1.224	1.832**	0.895	2.046	6.246
**Public health insurance**	−0.552**	0.217	−2.546	0.576	−0.364	0.277	−1.315	0.695
**Age**	−0.022***	0.008	−2.704	0.979	−0.030****	0.009	−3.357	0.970
**Gender**	0.018	0.162	0.109	1.018	−0.093	0.159	−0.582	0.911
**Marital status**								
Married	REF	REF	REF	REF	REF	REF	REF	REF
Partnered	−0.350	0.338	−1.037	0.704	−0.064	0.386	−0.166	0.938
Separated	−14.850	1693.000	−0.009	0.000	−0.348	1.521	−0.229	0.706
Divorced	0.664	0.671	0.989	1.942	0.185	0.834	0.222	1.203
Widowed	0.296	0.223	1.324	1.344	0.052	0.215	0.243	1.053
Never married	1.092**	0.548	1.995	2.980	1.675	1.094	1.531	5.339
**Household size**	−0.052	0.059	−0.878	0.949	0.077	0.061	1.273	1.080
**Education**								
Less than lower secondary	REF	REF	REF	REF	REF	REF	REF	REF
Upper secondary and vocational training	0.039	0.327	0.118	1.039	−0.714**	0.306	−2.336	0.490
Tertiary	−14.300	551.500	−0.026	0.000	−1.015	0.690	−1.471	0.362
**“Hukou” type**								
Agricultural “hukou”	REF	REF	REF	REF	REF	REF	REF	REF
Non-agricultural “hukou”	−0.161	0.234	−0.690	0.851	−0.066	0.219	−0.300	0.936
Unified residence “hukou”	0.269	0.597	0.451	1.309	−2.380**	1.188	−2.004	0.093
Do not have “hukou”	–	–	–	–	14.090	624.100	0.023	1,315,858.681
**Income**	0.000 ***	0.000	−2.662	1.000	0.000***	0.000	−2.764	1.000
**Health status**								
Excellent	REF	REF	REF	REF	REF	REF	REF	REF
Very good	14.340	1281.000	0.011	1,689,595.991	−14.670	624.200	−0.024	0.000
Good	14.750	1281.000	0.012	2,545,913.290	−14.630	624.200	− 0.023	0.000
Fair	14.250	1281.000	0.011	1,544,174.467	−14.540	624.200	−0.023	0.000
Poor	14.740	1281.000	0.012	2,520,581.029	−14.180	624.200	−0.023	0.000
**Functional limitation**	0.378 **	0.169	2.240	1.460	0.289*	0.173	1.667	1.335
**Number of chronic diseases**	0.142 ****	0.041	3.496	1.153	0.060	0.040	1.477	1.062
McFadden R^2^	9.270%				6.766%			
AUC	0.647				0.652			

*p < 0.10; **p < 0.05; ***p < 0.01; ****p < 0.001

Table 5 reports the results of binary logistic regression where specific types of public and private health insurance were included as the independent variables. It was found that UEI (OR: 0.11; 95% CI: 0.03–0.30) and NRCI (OR: 0.71; 95% CI: 0.51–1.01) were negatively associated with outpatient financial strain. The same negative influence of UEI on inpatient financial strain was reported (OR: 0.50; 95% CI: 0.25–0.96). Similarly, SPI enrollees were found to have 13.52 times the odds (95% CI: 1.67–416.14) of having inpatient financial strain, relative to those without SPI. Meanwhile, age, marital status, education, “hukou” type, income, functional limitation, and number of chronic diseases were statistically associated with outpatient or inpatient financial strain. The McFadden’s *R*^*2*^ of the outpatient and the inpatient regression model was 8.58 and 7.38%, respectively. Besides, both the outpatient (AUC = 0.70) and the inpatient (AUC = 0.65) regression model were found to have moderate predictive power.

**Table 5:** The impacts of different types of health insurance on financial strain for people with chronic diseases

	***Outpatient financial strain***				***Inpatient financial strain***			
	***Estimate***	***Std.Error***	***Z-value***	***OR***	***Estimate***	***Std.Error***	***Z-value***	***OR***
Intercept	−15.130	1254.000	−0.012	0.000	15.980	624.200	0.026	8,710,153.743
**UEI**	−2.221****	0.571	−3.892	0.109	−0.701**	0.341	−2.058	0.496
**URI**	−0.082	0.384	−0.213	0.921	0.038	0.404	0.095	1.039
**NRCI**	−0.338*	0.174	−1.942	0.713	−0.288	0.196	−1.465	0.750
**EPI**	1.207	1.246	0.969	3.343	14.620	882.700	0.017	2,235,554.827
**SPI**	−1.323	1.084	− 1.220	0.266	2.604**	1.308	1.991	13.518
**Age**	−0.019**	0.008	−2.360	0.981	−0.028***	0.009	−3.085	0.972
**Gender**	0.043	0.163	0.264	1.044	−0.089	0.160	−0.554	0.915
**Marital status**								
Married	REF	REF	REF	REF	REF	REF	REF	REF
Partnered	−0.422	0.338	−1.249	0.656	0.000	0.385	−0.001	1.000
Separated	−14.920	1693.000	−0.009	0.000	−0.304	1.618	−0.188	0.738
Divorced	1.228	0.758	1.619	3.414	0.205	0.825	0.249	1.228
Widowed	0.288	0.225	1.278	1.334	0.029	0.216	0.134	1.029
Never married	1.010*	0.550	1.836	2.746	1.656	1.095	1.513	5.238
**Household size**	−0.059	0.059	−0.999	0.943	0.081	0.061	1.327	1.084
**Education**								
Less than lower secondary	REF	REF	REF	REF	REF	REF	REF	REF
Upper secondary and vocational training	0.310	0.340	0.912	1.363	−0.650**	0.314	−2.072	0.522
Tertiary	−14.110	517.400	−0.027	0.000	−0.908	0.700	−1.298	0.403
**“Hukou” type**								
Agricultural “hukou”	REF	REF	REF	REF	REF	REF	REF	REF
Non-agricultural “hukou”	0.096	0.307	0.312	1.101	−0.025	0.279	−0.089	0.975
Unified residence “hukou”	0.354	0.613	0.578	1.425	−2.513*	1.287	−1.952	0.081
Do not have “hukou”	–	–	–	–	13.950	621.200	0.022	1,143,952.581
**Income**	0.000***	0.000	−2.681	1.000	0.000***	0.000	−2.897	1.000
**Health status**								
Excellent	REF	REF	REF	REF	REF	REF	REF	REF
Very good	14.710	1254.000	0.012	2,446,086.602	−14.390	624.200	−0.023	0.000
Good	15.020	1254.000	0.012	3,335,055.904	−14.350	624.200	−0.023	0.000
Fair	14.530	1254.000	0.012	2,043,143.273	−14.260	624.200	−0.023	0.000
Poor	14.990	1254.000	0.012	3,236,490.106	−13.890	624.200	−0.022	0.000
**Functional limitation**	0.363**	0.170	2.141	1.438	0.271	0.174	1.555	1.311
**Number of chronic diseases**	0.143****	0.041	3.434	1.154	0.052	0.040	1.287	1.053
McFadden R^2^	8.580%				7.379%			
AUC	0.700				0.653			

*p < 0.10; **p < 0.05; ***p < 0.01; ****p < 0.001

Table 6 reports the regression results after adjusting for endogeneity. It was found that public health insurance (OR: -0.48, 95% CI: 0.25–0.90) was still negatively associated with having outpatient financial strain. Our study fails to obtain a matched inpatient sample because only 11 out of 831 people had private health insurance.

**Table 6:** The impacts of health insurance on financial strain for people with chronic diseases (adjusting for endogeneity)

	***Outpatient financial strain***				
	***Estimate***	***Std.Error***	***Z-value***	***OR***	***Matched pairs*** ***(Sample size)***
Public insurance	−0.741**	0.329	−2.255	0.476	142 (284)
Private insurance	–	–	–	–	–
URCI	–	–	–	–	–
NRCI	−0.279	0.250	−1.118	0.756	318 (636)
EPI	–	–	–	–	–
SPI	–	–	–	–	–

*p < 0.10; **p < 0.05; ***p < 0.01; ****p < 0.001

## Discussion

Our research employed binary logistic regression technique to explore the impacts of different health insurance types on inpatient and outpatient financial strain. After controlling for a range of covariates, we demonstrated that public health insurance was expected to reduce the outpatient financial barriers faced by people with chronic diseases, while private health insurance was found to be a positive predictor of inpatient financial barriers.

Our research demonstrated that only public health insurance had the potential to reduce the financial burden for people with chronic diseases, which is consistent with previous studies [19, 37]. The positive effects of public health insurance on reducing financial strain could be explained by that public health insurance is with a relatively bigger pool and hence has higher potential for financial risk protection. Specifically, our research demonstrated that public health insurance was efficient in reducing the outpatient financial strain, while was not statistically associated with the reduction of inpatient financial strain. Furthermore, we found that UEI was the only public health insurance that was expected to reduce both outpatient and inpatient financial barriers, which aligns with previous research [20, 21]. UEI is mandatory and has higher insurance premiums, which may explain why UEI was more efficient in reducing financial strain than URI and NRCI. Meanwhile, another possible reason lies in that although the Chinese government has been increasing financial input on developing NRCI [38], people covered by NRCI are rural residents who tend to have lower economic status and are more vulnerable to the economic burden of chronic diseases.

Our research also demonstrated that the private health insurance was a positive predictor of having inpatient financial strain. As stated above, public health insurance was ineffective in reducing the inpatient financial strain for people with chronic diseases. In such circumstance, people with chronic diseases may in turn purchase private health insurance to relieve potential financial problems incurred by chronic diseases. Moreover, people with chronic diseases are more likely to purchase private health insurance given that they are more cognizant of their own health conditions [39], and the existence of adverse selection in private health insurance market may explain why private insurance was positively associated with financial strain.

Several policy implications were derived based on our research findings: First, strategies targeting at prevention and treatment of chronic diseases should be further developed [40]. For instance, policies such as the “Medium- and long-term plan for the prevention and treatment of chronic diseases in China (2017-2025)” [41] were suggested to be further diffused and associated measures were suggested to be proposed to facilitate the implementation of these health insurance policies, with the goal of alleviating the financial difficulty faced by people with chronic diseases. Second, the capacity of public health insurance in reducing the inpatient financial barriers for people with chronic diseases should be improved. People with chronic diseases usually suffer from problems in the physical, psychological and social domains, having diverse and complex needs in the areas of prevention, care, and cure [42]. Therefore, public health insurance schemes in China are suggested to shift from focusing on medical treatment to covering a wide range of healthcare services. Meanwhile, more efforts should be put on improving the financial protective ability of public health insurance in China. Third, an integrated health insurance system is suggested to be constructed, given that health insurance schemes in China differ distinctively in terms of their premiums and the reimbursement ratios and weakened the mutual aid of the health insurance system [43]. Forth, exploring the potential of private health insurance in mitigating the financial barriers for people with chronic disease should be set as another policy priority. A prior study has demonstrated that in the case of unsubscribed private health insurance among the people with chronic diseases, the unmet medical experience was significantly higher than that of those who signed up [18]. This finding highlighted the paramount significance of private health insurance in satisfying the medical needs of people with chronic diseases. However, private health insurance only accounted for a minor part in the health insurance market in China [44], and there is an urgent need for further exploring the potential effects of private health insurance in assisting people with chronic diseases to access affordable health services.

Our research has some strengths: first, our research has the potential to provide both practical and academic implications. We demonstrated that public health insurance was useful in helping people with chronic diseases to access affordable outpatient health services, while private health insurance may serve as a positive predictor of having inpatient financial strain. These results may inspire researchers and policymakers to re-consider the role and function of different types of health insurance in preventing and managing chronic diseases in China. Second, our study used a national-level dataset and therefore is representative of China.

However, several limitations should be recognized: Limited by data accessibility, we were unable to measure the magnitude of financial strain for specific types of chronic diseases. Nevertheless, our research was still useful in predicting the impacts of health insurance on financial barriers of people with chronic diseases. Second, only a small number of our sampled participants were separated, did not have “hukou”, and had tertiary education, which resulted in large standard errors and odds ratios. Future studies are suggested to include more participants to evaluate the impacts of these categories on financial strain. Another limitation was that we expected that the endogenous relationship between financial strain and purchasing health insurance may bias our estimation results. Our study adjusted endogeneity for the outpatient sample, but failed to solve the possible endogenous relationship between having private insurance and inpatient financial strain. Nevertheless, we believe that our research findings could provide preliminary evidence that for people with chronic diseases, having private insurance was positively associated with inpatient financial strain. We highly recommended future research to employ appropriate instrumental variables or other econometric techniques to generate more precise estimation results.

## Conclusion

This study demonstrates that public health insurance was expected to reduce the outpatient financial strain, while has no statistically significant influence on alleviating the inpatient financial strain for people with chronic diseases. This work highlighted that private health insurance was a positive predictor of having inpatient financial strain for people with chronic diseases in China. A wide range of healthcare services are suggested to be covered by public health insurance and the protective ability of public health insurance in solving the inpatient financial strain for people with chronic diseases. Additionally, further exploration on the potential of private health insurance on reducing the financial strain for people with chronic diseases should be warranted.

## Data Availability

The data that support the findings of this study are available from CHARLS, which are available on the website: http://charls.pku.edu.cn/pages/about/charls/en.html.

## Abbreviations

NCDs: Non-communicable diseases
CHARLS: China Health and Retirement Longitudinal Study
UEI: Urban Employee Basic Medical Insurance
NRCI: New Rural Cooperative Medical Insurance
URI: Urban Resident Basic Medical Insurance
EPI: Employer-paid private insurance
SPI: Self-paid private insurance
CNY: Chinese Yuan
AUC: Area Under the Curve
OR: Odds ratio
CI: Confidence interval
